# The urgency of cautious prescription for fluoride toothpaste: Recommendations for the stakeholders

**DOI:** 10.1016/j.jtumed.2023.09.008

**Published:** 2023-10-12

**Authors:** Rizwan Ullah, Syed K. Iqbal, Muhammad S. Zafar

**Affiliations:** a Department of Oral Biology, Jinnah Sindh Medical University, Karachi, Pakistan; b Department of Dental Materials Science, Jinnah Sindh Medical University, Karachi, Pakistan; c Department of Restorative, Dentistry, Taibah University, Almadinah Almunawwarah, KSA; d School of Dentistry, University of Jordan, Amman 11942, Jordan; e Department of Dental Materials, Islamic International Dental College, Riphah International University, Islamabad, Pakistan

Fluoride is one of the most abundant elements present on the earth. It is present naturally in water, soil, animals, and plants. The fluoride ions have proven antimicrobial and remineralizing properties gained through the following key mechanisms^1^:

1. Fluoride inhibits the growth of the microbes and their metabolism by enzyme inhibition such as the reduced formation of IgA protease.
2. Promotion of remineralization by the formation of the fluorapatite mineral phase. This phase is more resistant to acid demineralization as compared to hydroxyapatite.
3. Reduction in extracellular polysaccharide production which reduces the bacterial attachment to tooth tissues.

Due to these valuable properties, fluoride is incorporated into a range of products such as water, food and beverages, oral hygiene products, and dental materials. Fluoride is also delivered into the oral cavity through special devices.^1^^,^^2^ On the other hand, the excess consumption of fluoride may lead to various harmful effects. A few examples of detrimental effects are dental and skeletal fluorosis, hypersensitivity, gastric irritation, renal system insufficiency, numbness, and muscular spasms.^3^ Due to fluoride's beneficial and adverse effects, it is one of the most widely researched topics in dentistry and several highly cited manuscripts have been published highlighting the effects of fluoride on oral health.^4^ Through this scientific communication, we aim to summarize the existing reports of dental fluorosis, fluoride toothpaste availability, and recommendations for cautious prescription of fluoride-containing oral hygiene products.

## Status of dental fluorosis and fluoride toothpaste availability in Pakistan and KSA

The excess daily consumption of fluoride (1 mg/l or 0.1 mg/kg) during tooth formation leads to dental fluorosis. Dental fluorosis may range from mild fluorosis in which some faint white lines or steaks are visible under natural light to severe form of fluorosis in which there are esthetic concerns due to the disfigurement of teeth.^5^ Naturally or artificially fluoridated water and toothpaste are the two main sources of fluoride. There are numerous reports of dental fluorosis in Pakistan and KSA due to excessive ingestion of fluoride (Table 1).

**Table 1:** Studies reporting the prevalence of dental fluorosis in Pakistan and KSA.

Study	Study settings	Study participants	Prevalence and severity	Possible fluoride exposure
Sami et al.,^6^	Quetta, Pakistan	School children Age 12 years	Prevalence: 63% Moderate fluorosis: 50.4%	Tube well water.
Khan et al.,^7^	Peshawar, Pakistan	Population age 11 years onwards	Prevalence: 40% Questionable: 11.30% Very Mild: 15% Mild: 8.4%	Tap water, treated water, well water.
Mohsin et al.,^8^	Karachi, Pakistan	School children age 6–15 years.	Prevalence: 53.33% Questionable: 13.3% Very mild: 25.7% Mild: 20.95%	Groundwater.
Das et al.,^9^	Asir Region, KSA	Population age 9–50 years	Prevalance: 20% Questionable: 29% Very Mild: 12.2% Mild: 6.6%	Bottled water, filtered water, well water.
Khan et al.,^10^	Dammam, KSA	School children 6–12 years	Prevalence: 33% Mild and Moderate: 69%	Not discussed.
Alhobeira et al.,^11^	Hail, KSA	Population age 10 years onwards	Prevalence: 73.5%. Mild to moderate forms	Mineral water, tap water, community water source, well water.

Despite the prevalence and risk of dental fluorosis our study regarding the availability of pediatric fluoride toothpaste in Pakistan and KSA indicates that fluoride-containing toothpastes are easily available over the counter. The fluoride concentration in these toothpastes ranges from 500 to 1450 ppm fluoride in Pakistan and 250 to 1450 ppm fluoride in KSA.^12^

## Recommendations to the stakeholders

The following are some of the recommendations related to the safe prescription use of fluoridated toothpaste:

1. There is a need for curriculum reform because as compared to the curriculums of American and European dental schools in KSA, only a few dental schools reported offering dental cariology as a stand-alone course. According to our search, no data was available for Pakistan.^13^, ^14^, ^15^
2. In countries with fluoride toxicity, the high concentration fluoride toothpastes are available over the counter. There should be strict regulations and these toothpaste should only be available unless it is prescribed by a registered dental professional.^12^
3. Children living in a highly fluoridated area are at risk of toxicity especially if they are exposed to other sources of fluoride. Therefore, low or fluoride-free toothpaste should be prescribed. The children should be supervised by parents or guardians during tooth brushing to ensure that they are using an appropriate amount of toothpaste (pea size) and are not ingesting the toothpaste because of the developing swallowing reflex or toothpaste flavor.^12^^,^^16^^,^^17^ The manufacturer of fluoride toothpaste should provide complete visual and written instructions on the packaging.^17^
4. In populations at risk of fluoride toxicity, other factors contributing to dental caries should be addressed, for example by providing basic dental care, oral health education, and dietary modifications.^5^

## Conclusions

The authors would like to emphasize that dental caries is a multifactorial disease, which is not caused merely by fluoride deficiency. Furthermore, fluoride supplements also do not reverse active and gross carious lesions. These fluoride-containing products may cause toxicity in individuals who are already exposed to fluoride from dietary and environmental sources. Therefore, for the population at risk of dental caries and fluoride toxicity, other remedial measures beyond the use of fluoride such as patients, parents, or caregivers education and counseling about oral hygiene maintenance, and food selection should be the main focus.

## Source of funding

This research did not receive any specific grant from funding agencies in the public, commercial, or not-for-profit sectors.

## Conflict of interest

The authors have no conflict of interest to declare.

## Ethical approval

Ethical approval is not required for the letter to the editor.

## Authors contributions

RU and SMKI conceived the idea. RU wrote the initial draft of the article. MSZ and SMKI wrote a part of the article and critically revised the final draft. All authors have critically reviewed and approved the final draft and are responsible for the content and similarity index of the manuscript.
